# Inhibition of miR-652-3p Regulates Lipid Metabolism and Inflammatory Cytokine Secretion of Macrophages to Alleviate Atherosclerosis by Improving TP53 Expression

**DOI:** 10.1155/2022/9655097

**Published:** 2022-10-07

**Authors:** Haiyun Liu, Changpeng Zuo, Lijuan Cao, Naiquan Yang, Tingbo Jiang

**Affiliations:** ^1^Department of Cardiovascular, The First Affiliated Hospital of Soochow University, Suzhou 215000, China; ^2^Department of Cardiovascular, The Affiliated Huai'an Hospital of Xuzhou Medical University, Huai'an, Jiangsu 223002, China

## Abstract

**Purpose:**

The aim was to elucidate the regulatory function of miR-652-3p on lipid metabolism and inflammatory cytokine secretion of macrophages in atherosclerosis.

**Methods:**

miR-652-3p level in atherosclerosis patients, ox-LDL-treated macrophages, and their controls were monitored by Q-PCR. After ox-LDL treatment and miR-652-3p mimic, si-TP53 and their controls transfection, ELISA, and Q-PCR assays were used to detect IL-1ß, IL-6, and TNF-*α* levels. oil red O staining was processed to verify cholesterol accumulation. CE/TC and lipid metabolism were also detected. The protein levels of ABCA1, ABCG1, PPAR*α*, CRT1, ADRP, and ALBP were detected by western blot assay. Based on the TargetScan database, the TP53 3′UTR region had complementary bases with miR-652-3p, which was also verified by dual-luciferase reporter gene assay. Finally, the regulation of miR-652-3p and TP53 was confirmed by rescue assay in atherosclerosis.

**Results:**

miR-652-3p is highly expressed in atherosclerosis, miR-652-3p inhibitor decreased IL-1*β*, IL-6, and TNF-*α* expression after ox-LDL treatment. Knockdown of miR-652-3p reduces foam formation in ox-LDL-treated macrophages. miR-652-3p inhibitor ameliorates cholesterol accumulation and lipid metabolism disorder. miR-652-3p negatively regulated TP53 in atherosclerosis. Si-TP53 rescued the effect of miR-652 inhibitor in atherosclerosis.

**Conclusion:**

miR-652-3p regulates the lipid metabolism of macrophages to alleviate atherosclerosis by inhibiting TP53 expression. It might be a potential target for atherosclerosis treatment.

## 1. Introduction

Atherosclerosis (AS) is a complex pathophysiological process caused by the formation of plaques that accumulate cholesterol on the arterial walls [1]. Common diseases caused by AS include CAD, MI, stroke, and abdominal aortic aneurysm (AAA) [2, 3]. Cardiovascular disease even leads to death [4]. With the aging of the population, the incidence of AS shows a clear trend of increasing.

The study of the pathogenesis of AS has been going on for nearly a century. Dysregulation of lipid metabolism activates the biological function of immune cells [5]. The formation of atherosclerotic inflammatory response is due to lipid activation of multiple signal transduction pathways related to inflammation and apoptosis [6]. In particular, multiple transcription factors such as NF-*κ*B, NFAT, and STAT1/3 are activated, and each transcription factor regulates multiple downstream genes related to inflammation, oxidative stress, and cell cycle regulation [7, 8]. Macrophages are a major contributor to AS pathogenesis and development. Most foam cells are formed from macrophages, and lipid metabolism disorders in macrophages are prerequisites for forming foam cells. Under normal circumstances, a dynamic balance is maintained between macrophages' lipid intake, ester hydrolysis, and outer row [9]. However, extracellular lipid levels cause intracellular metabolism abnormalities, and the expression of receptors and enzymes related to macrophages and lipid metabolism will change [10]. Ox-LDL-induced macrophage abnormal lipid metabolism and increased inflammation are important factors leading to atherosclerotic plaque progression [11]. Monocyte-derived macrophages can secrete a series of inflammatory factors after excessive lipid uptake [12, 13]. It induces a local chronic inflammatory response in coronary arteries, which in turn triggers the occurrence and progression of atherosclerotic plaques [14].

However, AS is a complex inflammatory response formed after the long-term action of multiple factors [15]. Therefore, studying the gene expression in AS will be the key to a deeper understanding of the pathophysiological mechanism of AS formation. The emergence of miRNA provides a great possibility for the realization of this goal. MiRNAs are abnormally expressed in the intimal lesions of AS and vascular occlusion [16, 17]. Ji et al. [18] detected many aberrantly expressed miRNAs in the neovascularization of the intima of mouse arteries injured by balloon catheters by microarray analysis. In addition, Liang et al. [19] demonstrated aberrant expression of some miRNAs, such as let-7 miRNA in human and murine noninjured atherosclerotic vessels.

Recently, miR-652-3p was referred to be a potential target for AS. For instance, Vegter et al. [20] verified that the expression of miR-652-3p was critical in AS and cardiovascular disease. Besides, Huang et al. [21] confirmed that miR-652-3p targeted cyclin D2, and further affect the endothelial repair and AS development. However, the function of miR-652-3p in AS is complicated. This study aimed to elucidate the role of miR-652-3p on AS by studying the effect of miR-652-3p on macrophage lipid metabolism in atherosclerotic plaques. It is hoped that it will play a role in promoting the research on the pathogenesis, diagnosis, prognosis, and efficacy judgment of AS.

## 2. Materials and Methods

### 2.1. Patients and Serum Sample Collection

A total of 60 cases were from patients treated in our hospital from June 2021 to June 2022. All cases are unrelated, and patients excluded by relevant examinations include acute and chronic infection, surgery, trauma, liver and kidney disease, malignant tumor, rheumatoid disease, secondary hypertension, heart failure, heart valve disease, and alcoholism. Based on coronary angiography, patients with less than 50% stenosis of major vessels were diagnosed with coronary atherosclerotic stenosis. All subjects fasted for more than 8 h, 4 mL of cubital venous blood was collected in the early morning, and the serum was separated for testing. The patients involved affix their signatures on informed consent.

### 2.2. Treatment of Macrophage Cells

The macrophage cells RAW264.7 were obtained from the ATCC (Manassas, VA). Cells were cultured in a carbon dioxide incubator with RPMI-1640 medium with 5% FBS. The cultured RAW264.7 was treated with ox-LDL (100 ms/L) for 3 h.

The miR-652-3p inhibitor and NC were designed and synthesized by Guangzhou Ribo Biological Company. Macrophages were divided into four groups, including control, ox-LDL, ox-LDL + NC inhibitor, and ox-LDL + miR-652-3p inhibitor. The transfection steps were performed according to the instructions of the Lipofectamine™ 2000 reagent (Invitrogen, Carlsbad, CA). miR-652-3p mimic, si-TP53, and their controls were also transfected by Lipofectamine™ 2000 reagent.

### 2.3. Q-PCR Assay

Total RNA was collected from samples in each group by Trizol reagent, and RNA was transcribed into cDNA. Q-PCR detection was performed by the SYBR Primix Ex Taq detection kit using cDNA as a template. The reaction system contains 2 *μ*L of cDNA, 10 *μ*L of 2 × SYBR Primix Ex Taq, 1 *μ*L of upstream and downstream primers, and 20 *μ*L of ddH_2_O. The reaction program was set at 95°C for 5 min, followed by 40 cycles (95°C for 30 s, 60°C for 20 s, and 72°C for 20 s). The experiment was carried out 3 times, Ct was calculated, and U6 and GAPDH were chosen as the reference. The miR-652-3p level was analyzed by the relative quantitative 2^−ΔΔCt^ method. The primer sequence information is shown in Table 1.

### 2.4. ELISA Assay

Cells from each group were collected in sterile tubes and centrifuged for 20 minutes, and the supernatant was carefully collected. PBS was used to dilute the cell suspension (10^6^ cells/mL) when detecting intracellular components. Repeated freezing and thawing were processed for releasing intracellular components. ELISA kits were purchased from Neobioscience Co., Ltd. (China). Double antibody ELISA was used to show IL-1*β*, IL-6, and TNF-*α* levels in the supernatant according to the kit instructions. The OD_450_ was determined for each sample. A standard curve was drawn, and the protein expression is calculated according to the standard curve.

### 2.5. Detection of Foam Cell Formation

The 15 mm sterile slides were placed in a 12-well plate in advance and the cell count was 1 × 10^6^ mL^−1^ After intervention for 72 h, the samples were washed, fixed with 4% paraformaldehyde for 10 min, soaked in 60% ethanol for 1 min, and treated with oil red O staining solution. The samples were rinsed and stained with hematoxylin for 5 min. After the slides were rinsed and dried, they were sealed with glycerin gelatin and observed under an oil microscope (Olympus).

### 2.6. Detection of Lipid Metabolism

The amplex red cholesterol detection kit was used for detection. To determine total cholesterol (TC) and free cholesterol (FC), cells were obtained by chloroform/methanol extraction (2 : 1 by volume). The chloroform phase layers were collected, dried, and then stayed in the reaction buffer. The content of cholesteryl ester (CE) was calculated by measuring TC and FC content in each sample. TC and FC were detected with an automatic biochemical analyzer (Beckman). CE/TC values were applied to assess lipid metabolism.

### 2.7. Western Blot Assay

Cells in each group were collected, NP-40 lysate was added, and the total protein in cells was extracted on ice. SDS-PAGE gels were prepared, and equal amounts of protein were taken for electrophoresis. After the protein was separated, it was transferred to the PVDF membrane; the sample was soaked in 5% skimmed milk for blocking for 1 h, and the membrane was washed. After the corresponding primary antibody (ABCA1, ABCG1, PPAR*α*, CRT1, ADRP, and ALBP, 1 : 1000, Abcam) was added, the samples were then incubated on a vertical shaker at 4°C for 10 h. After washing, a secondary antibody diluted 1 : 3000 was applied for incubation at room temperature for 2 h. ECL luminescent solution was added to the sample, followed by the development, exposure, and image acquisition. *β*-Actin was normalized as a reference, and the Quantity One software was used to analyze the data.

### 2.8. Dual-Luciferase Reporter Gene Assay

TargetScan online database predicted that the TP53 3′UTR region had complementary bases with miR-652-3p. According to the predicted results, TP53 wild-type (TP53-Wt) and TP53 mutant (TP53-Mut) luciferase recombinant vectors were constructed, respectively. Cells were seeded into 24-well plates at 5 × 10^4^ cells/well and cultured in a 37°C incubator. The cells were divided into NC + TP53 − Wt, miR-652-3p mimic + TP53 − Wt, NC + TP53 − Mut, and miR-652-3p mimic + TP53 − Mut groups. The transfection procedure was performed according to the instruction manual of Lipofectamine 2000 transfection reagent and incubated for 2 d for reaction. A dual-luciferase reporter gene detection kit was used. The relative luciferase activity of cells in each group was calculated by normalizing the activity of Renilla luciferase.

### 2.9. Statistical Analysis

Statistical analysis was undertaken by SPSS 21.0 software. The data were shown as mean ± SD. The *t*-test and the one-way analysis were chosen for two and multiple groups, respectively. Statistically significant was with *P* < 0.05.

## 3. Results

### 3.1. miR-652-3p Is Highly Expressed in AS

**Figure 1:**
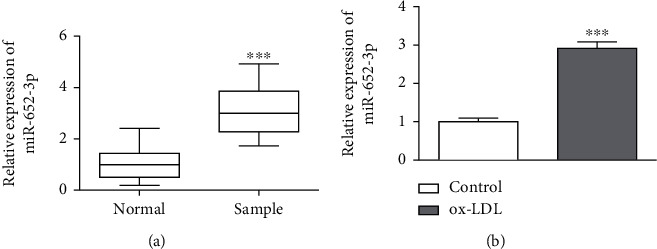
miR-652-3p expressed highly in atherosclerosis samples. (a) miR-652-3p was highly expressed in AS patients. (b) miR-652-3p was significantly higher in the ox-LDL group. ^∗∗∗^*P* < 0.001.

Q-PCR assay was processed to detect the miR-652-3p expression in serum samples, ox-LDL-treated macrophage cell lines, and their controls. As shown in Figure 1(a), miR-652-3p was highly expressed in AS patients. Similar results were also obtained in the cell experiment. After treatment, miR-652-3p was higher than that in the control (Figure 1(b)). Thereby, miR-652-3p was critical in the pathogenesis of AS.

### 3.2. Knockdown of miR-652-3p Attenuates Inflammation in Ox-LDL-Treated Macrophages

**Figure 2:**
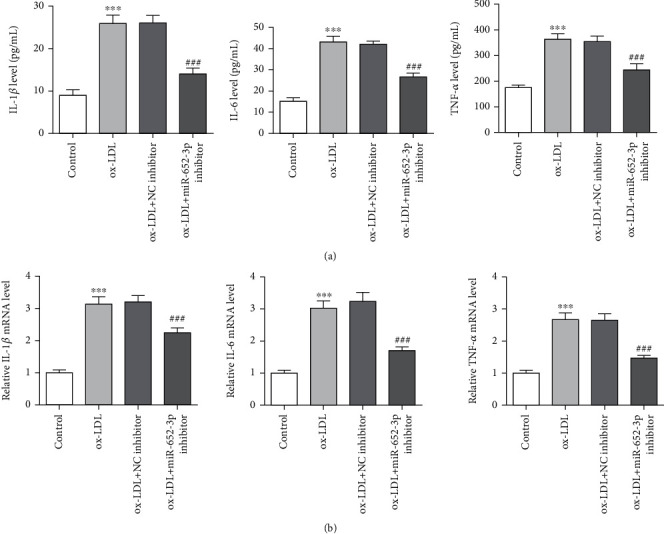
Knockdown of miR-652-3p attenuates inflammation in ox-LDL-treated macrophages. (a) Q-PCR assay for detecting IL-1ß, IL-6, and TNF-*α* levels. (b) ELISA assays were undertaken to detect the expression of immune factors vs. control group, ^∗∗∗^*P* < 0.001 vs. ox-LDL + NC inhibitor group, *###P* < 0.001.

Macrophages could contribute to local inflammation by producing proinflammatory cytokines in AS [22]. ELISA and Q-PCR assays were undertaken to detect immune factors, including IL-1*ß*, IL-6, and TNF-*α*. In vivo, IL-1 is mainly responsible for the acute response. Cytokines of the IL-1 family were also a part of the host to resist infection [23]. Besides, TNF-*α* is a cytokine involving systemic inflammation, which is mainly secreted by macrophages [24]. Conversely, IL-6 systematically acts on the liver to produce acute proteins, such as CRP, fibrinogen, and osteoclast activation inhibitors [25]. The levels of IL-1*β*, IL-6, and TNF-*α* were higher in the ox-LDL group and ox-LDL + NC inhibitor group. The levels of IL-1*β*, IL-6, and TNF-*α* were lower in the DL + miR-652-3p inhibitor group (Figure 2(a)). The results of ELISA and Q-PCR assay were consistent. The ELISA assay showed that ox-LDL accelerated IL-1ß, IL-6, and TNF-*α* levels, while miR-652-3p knockdown reduced these proinflammatory cytokines (Figure 2(b)). According to the evidence, the knockdown of miR-652-3p reduced inflammation in ox-LDL-treated macrophages.

### 3.3. Knockdown of miR-652-3p Reduces Foam Cell Formation in Ox-LDL-Treated Macrophages

**Figure 3:**
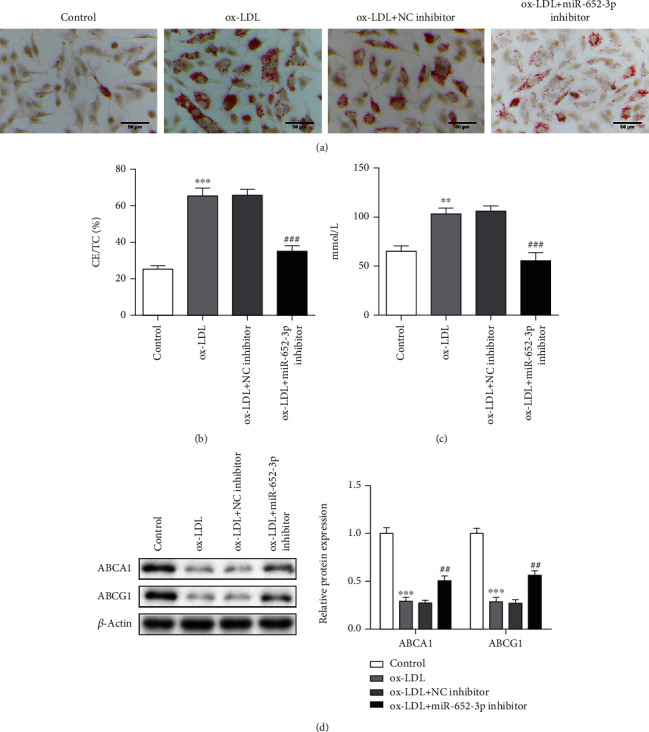
Knockdown of miR-652-3p reduces foam cell formation in ox-LDL-treated macrophages. (a) Oil red O staining. (b) The intracellular CE/TC ratio. (c) The cholesterol level of ox-LDL. (d) Western blot was processed to show ABCA1 and ABCG1 levels vs. control group, ^∗∗∗^*P* < 0.001 vs. ox-LDL + NC inhibitor group, ###*P* < 0.001.

The formation of foam cells often occurs early in the early stage of atherosclerosis [26]. The blood cells of patients with atherosclerosis have differentiated under the endometrium, forming macrophages, and devouring a large amount of low-density cholesterol, forming foam cells [27]. Interestingly, ox-LDL significantly increased foam cell formation in macrophages, while the knockdown of miR-652-3p reduced it (Figure 3(a)). The intracellular CE/TC value and cholesterol level of the ox-LDL treatment group were higher. miR-652-3p inhibitor decreased CE/TC value and cholesterol level (Figures 3(b) and 3(c)). ABCA1 and ABCG1 are two important proteins for cholesterol transport. ABCA1 is mainly responsible for promoting cholesterol efflux from cells to lipid-poor apoA-I, and ABCG1 is mainly responsible for promoting cholesterol efflux to mature high-density lipoprotein particles. At the same time, the two also interact to jointly promote reverse cholesterol transport. According to the western blot assay, ABCA1 and ABCG1 levels significantly decreased in the ox-LDL group, while they were upregulated after the miR-652-3p inhibitor was transfected (Figure 3(d)). Based on these results, miR-652-3p inhibitor reduces cholesterol accumulation in ox-LDL-treated macrophages.

### 3.4. miR-652-3p Inhibitor Reduces Lipid Metabolism Disorder in Ox-LDL-Treated Macrophages

**Figure 4:**
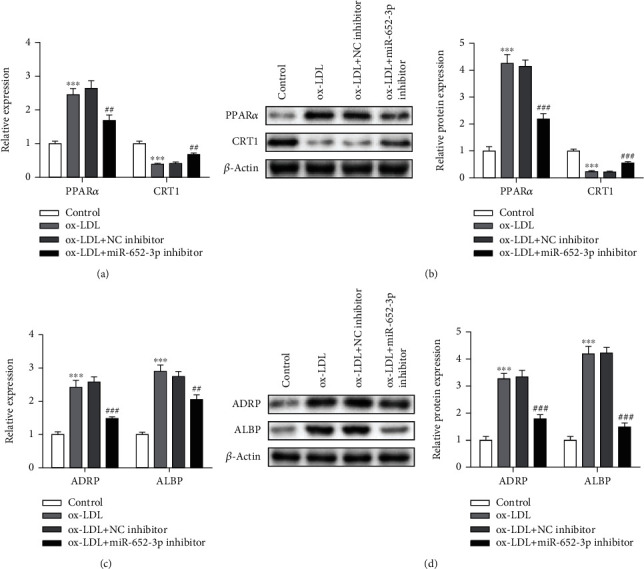
Knockdown of miR-652-3p reduces lipid metabolism disorder in ox-LDL-treated macrophages. (a) Q-PCR assay for detecting PPAR*α* and CRT1 levels. (b) Western blot assay for PPAR*α* and CRT1 protein expressions. (c) Q-PCR assay for detecting ADRP and ALBP levels. (d) Western blot assay for ADRP and ALBP protein expressions vs. control group ^∗∗∗^*P* < 0.001 vs. ox-LDL + NC inhibitor group, ##*P* < 0.01, ###*P* < 0.001.

PPAR*α*, CRT1, ADRP, and ALBP were biomarkers of lipid metabolism disorder. We performed Q-PCR and western blot to detect their expression. Interestingly, PPAR*α*, ADRP, and ALBP expressed higher in ox-LDL groups, while they expressed lower in the ox − LDL + miR − 652 − 3p inhibitor group. However, CRT1 showed an opposite trend (Figures 4(a)–4(d)). Thereby, miR-652-3p inhibitor reduces lipid metabolism disorder in ox-LDL-treated macrophages.

### 3.5. miR-652-3p Negatively Regulates TP53 Expression in AS

**Figure 5:**
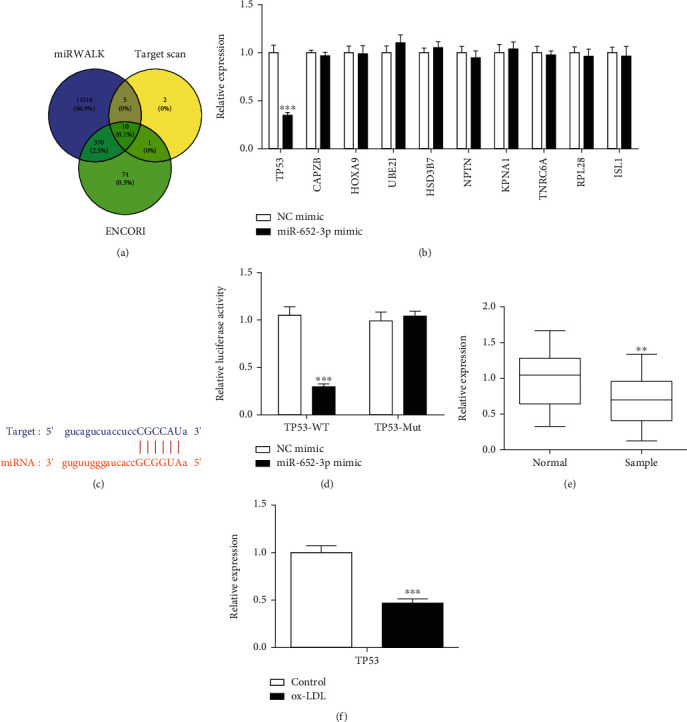
MiR-652-3p targets TP53 in AS. (a) Based on the database of miRWalk, TargetScan, and ENCORI online databases, a total of 10 crossover target genes were obtained. (b) Q-PCR was undertaken to detect the levels of TP53, CAPZB, HOXA9, UBE2I, HSD3B7, NPTN, KPNA1, TNRC6A, RPL28, and ISL1. (c) The binding site between miR-652-3p and TP53. (d) miR-652-3p mimic could reduce TP53 level in the wild group but did not affect the mutant group. (e) TP53 was expressed lower in AS serum samples. (f) After ox-LDL treatment, TP53 expressed lower in macrophages. ^∗∗^*P* < 0.01, ^∗∗∗^*P* < 0.001.

Based on the miRWalk, TargetScan, and ENCORI online databases, a total of 10 crossover target genes were obtained, including TP53, CAPZB, HOXA9, and UBE2I, HSD3B7, NPTN, KPNA1, TNRC6A, RPL28, and ISL1 (Figure 5(a)). Q-PCR was undertaken to detect these genes. Interestingly, miR-652 mimic downregulated the expression of TP53, while no significant difference was found in the other 9 genes (Figure 5(b)). Therefore, TP53 would be downstream of miR-652. The binding site was predicted by the TargetScan database (Figure 5(c)). miR-652-3p mimic could reduce the expression of TP53 in the wild group but did not affect the mutant group (Figure 5(d)). Furthermore, TP53 in the serum of AS patients and controls was also detected by Q-PCR. TP53 was expressed lower in AS serum samples (Figure 5(e)). After ox-LDL treatment, TP53 was expressed lower in macrophages (Figure 5(f)). According to the evidence, TP53 was a target of miR-652-3p.

### 3.6. Si-TP53 Rescued the Effect of miR-652 Inhibitor in AS

**Figure 6:**
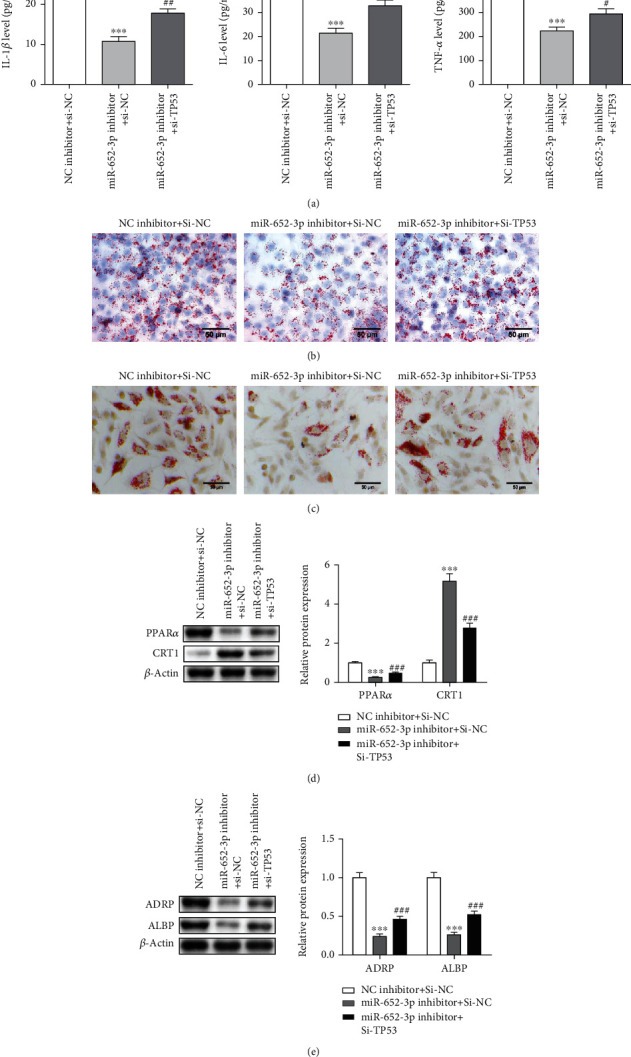
Si-TP53 rescued the effect of miR-652 inhibitor in AS. (a) miR-652-3p inhibitor decreased the expression of proinflammatory cytokines, while si-TP53 rescued the expressions. (b) Foam formation. (c) Cholesterol accumulation in ox-LDL-treated macrophages. (d) Western blot assay for PPAR*α* and CRT1 protein expressions. (e) Western blot assay for ADRP and ALBP protein expressions vs. NC inhibitor + siNC group, ^∗∗∗^*P* < 0.001 vs. miR-652-3p inhibitor + si-NC group, ###*P* < 0.001.

As the above results mentioned, miR-652-3p inhibitor decreased the levels of IL-1*ß*, IL-6, and TNF-*α*, while si-TP53 rescued their expression (Figure 6(a)). Besides, miR-652-3p inhibitor reduced foam formation and ameliorated cholesterol accumulation (Figures 6(b) and 6(c)). miR-652-3p inhibitor decreased PPAR*α*, ADRP, and ALBP expression, while accelerated CRT1 level (Figures 6(d) and 6(e)). It was worth noting that the function of miR-632-3p inhibitor was rescued when si-TP53 was cotransferred.

## 4. Discussion

AS is the main cause of various diseases, such as coronary heart disease [28]. Among the entire pathogenesis, lipid metabolism disorder is the pathological basis of AS [29]. In this study, we elucidated the regulatory mechanism of miR-652-3p on AS by studying the effect of miR-652-3p on macrophage lipid metabolism in vitro. Excitingly, we verified that miR-652-3p was highly expressed in AS. Moreover, it regulated the lipid metabolism of macrophages to participate in AS development via interacting with TP53.

In the previous study, miR-652-3p was confirmed to be expressed higher in various diseases, such as non-small-cell lung cancer [30], lymphoblastic leukemia [31], and cerebral ischemia [32]. Grimaldi et al. [33] researched TICAGROR for patients with acute coronary syndrome and referred that miR-652-3p expressed differently in the process of treatment. Moreover, miR-652-3p was verified to be involved in endothelial repair, atherosclerotic disease, and rehospitalizations [20, 21]. Interestingly, miR-652-3p inhibitor attenuates inflammation in ox-LDL-treated macrophages in this study. Besides, we also referred that the levels of ABCA1 and ABCG1 significantly decreased in the ox-LDL group, while they were upregulated after the miR-652-3p inhibitor was transfected. As we all know, ABCA1 is the main decisive factor in the level of blood high-density lipoprotein [34]. Therefore, the protein is particularly important in drug research and development for the treatment of arterial cell cholesterol deposition. ABCG1 is an ABC transfer protein located on the surface of the cell surface. In the plasma, HDL plays a critical role in promoting the flow of macrophage cholesterol outflow and atherosclerosis [35]. Besides, previous research shows that mice models lacking ABCG1 may cause cholesterol to accumulate a lot in macrophages and liver cells [36]. ABCG1 is highly expressed in macrophages, which is critical in the cholesterol reversal of macrophages [37]. Importantly, an imbalance of lipid homeostasis in macrophages in the early stages of arteriosclerosis leads to the accumulation of intracellular cholesterol, which in turn leads to foam cell formation [38]. The formation of cholesterol-rich foam cells is a key event in the early stages of AS [39]. In this study, the knockdown of miR-652-3p reduces foam formation in ox-LDL-treated macrophages. Furthermore, miR-652-3p inhibitor ameliorates cholesterol accumulation.

In addition to removing excess cholesterol in the body, ABCA1 and ABCG1 were also important in the suppression of inflammation, thus becoming critical targets for research on inhibiting AS progress [38]. Inflammation was an important immune defense mechanism of the body. Proinflammatory cytokines played a central role in infectious or noninfectious inflammatory diseases. Proinflammatory cytokines were mainly obtained by macrophages and involved in the upregulation of inflammatory responses. IL-1*β*, IL-6, and TNF-*α* were typical proinflammatory cytokines. These cytokines acted to contain and resolve inflammatory foci by activating local and systemic inflammatory responses. Based on the importance of these cytokines in the inflammatory response, we detected their expression and confirmed that miR-652-3p inhibitor attenuated inflammation via regulating inflammatory cytokines.

It was worth noting that miR-652-3p targeted TP53 expression in AS based on biological information analysis and molecular experiments in this study. The relationship between miR-652-3p and TP53 has not been reported so far. TP53 expression products were found in macrophages, which participated in the adjustment of macrophages during the process of AS [39]. Moreover, Guevara et al. [40] researched the role of TP53 in the occurrence and development of AS in vivo, confirming that lacking TP53 was related to the size of the AS lesion, macrophages, and lipoprotein. In this study, TP53 was confirmed to be the target of miR-652-3p. Interestingly, si-TP53 rescued the effect of the miR-652-3p inhibitor on lipid metabolism and inflammation. Thereby, the rescue assay further confirmed the role of miR-652-3p inhibitor and si-TP53 in the inflammatory and lipid metabolism of macrophages in AS.

In conclusion, miR-652-3p regulated lipid metabolism and inflammatory cytokine secretion of macrophages to alleviate AS by inhibiting TP53 expression. This study hopes to provide favorable clues to solve AS and related cardiovascular diseases through the miR-652-3p inhibitor's function in lipid metabolism and inflammatory cytokine secretion.

## Figures and Tables

**Table 1:** Primer sequence information.

Gene name	Forward primer (5′-3′)	Reverse primer (5′-3′)
miR-652-3p	AATGGCGCCACTAGGGTTGTG	CTCTACAGCTATATTGCCAGCCAC
TP53	CCAAACTGCTAGCTCCCATCA	GAAAGTAGGCCCTGGAGGATA
PPAR*α*	TGCCTTCCCTGTGAACTGAC	TGGGGAGAGAGGACAGATGG
CRT1	TCCCTGGTCATCACGGATCT	ATCGGCCGTTGAAGTTGGAT
TNF-*α*	CATGGAGGGGTTTGTCCGAA	AGAAATCGCAATTCATGTCGC
IL-6	CCCCAATTTCCAATGCTCTCC	CGCACTAGGTTTGCCGAGTA
IL-1*β*	TGCCACCTTTTGACAGTGATG	TTCTTGTGACCCTGAGCGAC
HOXA9	CCGGACGGCAGTTGATAGAG	CCAGCGTCTGGTGTTTTGTG
UBE2I	GGGAAGTCCCGAGACAAAGG	TGAGAAGGACGAGGGTAGGG
HSD3B7	GGACACAACCTCCTCCAGTG	CCTGCACGTTGACTTTGTGG
NPTN	CAGTGGTGGTACGCAGAAGT	ACCTCATCTGGCCTCTTCCT
KPNA1	GCCTGGAGCCGAAATCATGT	AGGTATGAGAGAGCCCAGCA
TNRC6A	TGCTGCATGTGAGAGTTCTTCT	CGGTCACTCAGCTTAACGGT
RPL28	CTTCCGCTACAACGGGCTAA	GAAGTGGCAGGTTTTCGCTG
ISL1	CGGTGCAAGGACAAGAAACG	GCAGGCTGATCTATGTCGCT
CAPZB	CCCCTGAACTGTCAGCTTGT	ATGGCCCCATCTTCCAAAGG
U6	CGCACTTTACGGCTACCTCT	GCGACAAGGGAAGGGAACAA
GAPDH	GCATCTTCTTGTGCAGTGCC	TACGGCCAAATCCGTTCACA

## Data Availability

All data generated or analyzed during this study are included in this article.
